# The Physical Activity and Sport Participation Framework—A Policy Model Toward Being Physically Active Across the Lifespan

**DOI:** 10.3389/fspor.2021.608593

**Published:** 2021-05-07

**Authors:** Hans Westerbeek, Rochelle Eime

**Affiliations:** ^1^Institute for Health and Sport, Victoria University, Melbourne, VIC, Australia; ^2^School of Science, Psychology and Sport, Federation University, Ballarat, VIC, Australia

**Keywords:** sport participation, physical literacy, physical activity, lifespan, ecosystem

## Abstract

The changing social and policy context in which sport is produced, delivered, and consumed is considered as a foundation for proposing a new integrated framework that incorporates participation in sport with participation in leisure-time physical activity (PA) more broadly. In order to position sport in the broader context of leisure-time PA, the concept of physical literacy is reviewed and integrated into the theoretical foundations of a new lifespan framework. It is argued that historically, sport policy largely focused on competitive club-based sport and elite performance and that in line with this, talent development pathway models were developed and implemented. However, with increasing physical inactivity globally, these models do not apply to the general population. This is why we propose a population-based “whole of sport ecosystem” lifespan model—the Physical Activity and Sport Participation (PASP) framework. We conclude that this framework may serve as a holistic policy and implementation guide for all in the sport ecosystem. This includes governmental PA and sport policy makers, sport governing bodies and clubs, and the ever-increasing range of private PA and sport providers and also health agencies. In recognition of the changing patterns of participation in PA and sport across the lifespan, the PASP framework can contribute to coordinated and integrated PA and sport policy development, which, in turn, can lead to strategies that tackle the global physical inactivity crisis.

## Introduction

The populations of developed but also developing nations are becoming increasingly inactive, and increasing proportions of populations do not meet physical activity (PA) guidelines. This has led to overweight and obesity becoming the leading risk factor for ill health in developed nations such as the USA and Australia (Australian Institute of Health Welfare, 2018). Physical inactivity is associated with various chronic diseases and early deaths (Ding et al., 2016). Increasing chronic disease and an aging population are putting extreme pressure on health costs with a conservative estimated cost of physical inactivity globally on the health care systems of (INT$) 54 billion in 2013 (Ding et al., 2016).

Modern *Homo sapiens* need to be more physically active, and this can be facilitated through a range of domains or contexts such as transport, home duties, work, and leisure time (Bauman et al., 2012). Three aspects specific to leisure-time PA (generally for choice and enjoyment) are type, mode, and setting (Eime et al., 2013). “Type” refers to activity (e.g., football, walking, and swimming), “modes” of participation can be in team sports (e.g., football, cricket, and netball), organized but non-competitive PA (e.g., cycling and running groups), individual sports (e.g., tennis, athletics, and triathlon), and non-organized or informal PA (e.g., going to the gym or going for a walk). Finally, one can participate in organizational settings (schools, clubs, or leisure centers), but also in neighborhood settings such as the street, the park, or at home (Eime et al., 2013).

There is increasing evidence that participation in sport can have health benefits above and beyond the physical, and that it can significantly improve mental and social health. More specifically, there is evidence that participation in organized sport can deliver improved health outcomes, largely due to the social nature of participation (Eime et al., 2013a,b). There is also evidence of a range of other health, behavior, and developmental benefits of participation in sport. This includes improved social interaction and self-esteem, fewer depressive symptoms (Eime et al., 2013b), better prosocial behavior, and less internalizing problems (Moeijes et al., 2018). Benefits of participation in sport for adults can include improved general well-being and reduced distress and stress (Eime et al., 2013). Furthermore, older adults can gain social health benefits with participation in sport providing a sense of belonging and opportunity to socialize (Jenkin et al., 2018). Historically, most sport participation models primarily focus on the journey that takes participants into elite sport. Very few, if any, models are population based and consider when, how, and who participates in community-level sport and non-organized recreational sport activities. Later in this paper, we will present some of the most prevalent theoretical models that have been used to describe phases and characteristics of sport participation, and how these models are focused on elite participation.

In addition to the wide-ranging health benefits of PA and more specifically sport, there has been recent attention internationally, to the role that PA can have on children's and adolescents' academic performance (Telford et al., 2012; Dyer et al., 2017; Castro-Sánchez et al., 2019). Academic performance is linked to PA as acknowledged in the current Australian national sports policy document, *Sport 2030* (Department of Health, 2018). It can be argued that the production and delivery of community and elite sport in many countries for long has existed in a vacuum, with little consideration of obvious connections to a larger industry, which includes parts of the health, education, and well-being sectors. Community and elite sport organizations have mostly been focused on delivering training and competition services to its membership, and as we will later explain, funded to increase participation numbers in sport.

Moore (1993) proposed in that regard that “successful [sport] businesses are those that evolve rapidly and effectively. Yet innovative [sport] businesses can't evolve in a vacuum. They must attract resources of all sorts, drawing in capital, partners, suppliers, and customers to create cooperative networks…. I suggest that a [sport organization] be viewed not as a member of a single industry but as part of a business ecosystem that crosses a variety of industries. In a business ecosystem, companies co-evolve capabilities…. They work cooperatively and competitively to support new products, satisfy customer needs” (pp. 75–86). Ever since Moore introduced the business ecosystem concept, there has been a move away from the vertically integrated “self-contained” corporation structure to highly dynamic networks of organizations in related industries that connect, collaborate, and depend on each other in order to create (more and better) shared value.

In this paper, we consider the changing social and policy context in which sport is produced, delivered, and consumed, as one of the drivers of change in regard to how sport organizations (can) produce value. This provides a foundation for developing a new integrated framework that incorporates participation in sport with participation in leisure-time PA at the population level more broadly. In a way, we are proposing to take an “ecosystem” approach to positioning sport as a form of leisure-time PA influenced by a wide spectrum of conditions and organizations that facilitate or inhibit people engaging in physical activities including sport. We also propose a lifespan approach because maintaining healthy PA levels remains crucial from cradle to grave. In order to position sport in the broader context of leisure-time PA, we will argue that the concept of physical literacy is foundational to this new framework.

It would be fair to say that Whitehead (2001) is the originator of the modern physical literacy concept [Bailey (2020) traces earlier use of the concept back to 1930]. Much of her foundational work from 2001, titled “The concept of physical literacy,” remains an important part of various attempts to fully and comprehensively define what is physical literacy. Whitehead (2001, 2010) notes the importance of realizing that “physical literacy” is a holistic concept and that it represents a lifelong journey, and that (movement) experiences and (motor) learning are vital in regard to becoming more physically literate. Physical education for school-aged populations in that regard remains an incredibly important platform for early development of physical literacy. Without sufficient (high quality) physical education, it will be hard to make up for this later in life when the capacity for learning new or enhancing existing motor skills diminishes. In 2013, Whitehead's definition had evolved to read that physical literacy “is the motivation, confidence, physical competence, knowledge, and understanding to value and take responsibility for maintaining purposeful physical pursuits/activities throughout the life course” (p. 65). However, there is no definitive global definition (yet) and there never may be given the fact that physical literacy is also (cultural) context dependent (Whitehead, 2010, 2013). As a matter of fact, authors such as Jurbala (2015), Edwards et al. (2018), and Bailey (2020) explain and review the multidimensional nature of the concept and question if there is sufficient definitional common ground for productive discussion and collaboration. We acknowledge that conceptualization of physical literacy remains ambiguous and that there is a paucity of empirical research. However, in their study identifying the utilization of physical literacy in the context of health and healh promition, Cornish et al. (2020) conclude that irrespective of lacking empirical evidence, “physical literacy may present a novel and holistic framework for health-enhancing physical activity interventions that consider factors vital to sustained participation in physical activity across the life course” (p. 1). We concur and feel that the concept itself offers tremendous scope for application to and integration with sport. We will explain our views by presenting some of the recent work in defining the concept. For the purpose of this paper, we will use Whitehead's (2013) definition, but it is useful to consider the recent work of Keegan et al. (2019) who defined physical literacy in the Australian context. In a modified Delphi study, the research team achieved consensus on a series of defining statements that read as follows:

- “The core of physical literacy—focused on the inherent potential of all humans to learn through physical interaction with the environment;
- Its constitution—based on integrated development spanning the four learning domains of physical, psychological, cognitive, and social;
- Its importance—in that physical literacy helps a person to learn more about the world, become more capable, and ultimately pursue a range of fulfilling activities, as well as the known benefits to health associated with PA; and finally
- The aspiration—describing a configuration, or possibly configurations, of this learning that becomes self-perpetuating, such that the individual persists with PA and movement pursuits, and/or reengages following interruptions such as injury, or significant life events.” (p. 112)

It is imperative to mention that each of the four domains noted above (physical, psychological, cognitive, and social) are made up of capabilities that develop physical literacy. These capabilities are referred to as elements and all can play a role (dependent on the individual) in developing or maintaining physical literacy. In the physical domain, there are 14 elements (such as strength, coordination, and agility), there are six elements in psychological (such as motivation and confidence), seven elements in social (such as collaboration and connectedness), and six elements in cognitive (such as awareness and rules). Having introduced the most recent theoretical advancements in regard to defining and operationalizing “physical literacy,” we can now position sport as a distinct and specific type of leisure-time PA as noted earlier in this paper. It has already been outlined that living a physically active life through participation in sport will have a significant positive impact on physical, social, and mental health, and on academic performance. Beyond this evidence, sport offers a highly visible and attractive (marketing) platform that can be used to engage people into leisure-time PA. For example, Craike et al. (2010) argued that when people enjoy the (physical) activity, they develop a preference for it and, hence, continue or increase their participation. Eime and Harvey (2018) argue that “fun” should be at the core of sport delivered through the community club-based system and that “fun” “is an integral component for participation at all ages. However, participation in sport is only fun if the program and environment is conducive to having fun, and if participants have an adequate level of skill to be competent and confident to play” (p. 25). This mainly relates to the constituting “physical” component of physical literacy, as introduced above.

Rather than focusing sport policies on simply increasing participation numbers annually, a shift to a more client-based focus that is zooming in on the offering or the development of sport activities that match skill levels with task challenges may well lead to increasing levels of joy in playing sport and as such a higher chance of retention. Competitive sport is not for everybody, as some sports are harder to master at a basic (more enjoyable) skill level than others. However, watching sport at the elite level and participating in sport from a young age presents significant opportunities to highlight and communicate the benefits of a physically active lifestyle. It also provides insightful examples of how an active lifestyle can be developed and maintained. As a matter of fact, the International Society for Physical Activity Health (2011) has identified sport as one of seven best buys to promote and encourage participation in PA.

The health (physical, mental, and social) and academic benefits that can be produced by participation in sport have significant economic ramifications. Shulenkorf and Siefken (2018) presented a sport-for-health model and argue the significant benefits that can be gained by using an integrated approach to health promotion and sport. In a way, they are arguing to consider sport production and health outcomes to be part of the same ecosystem. The Intergenerational Review of Australian Sport 2017, conducted by the Boston Consulting Group (2017), estimated that strong economic, health, education, and community benefits can be achieved by 2036 if Australia addresses the inactivity and obesity trend identified in the 2017 report. Westerbeek et al. (2019) report that these benefits include:

- “almost $12 billion in additional economic activity each year relative to what would be achieved without action;
- $2.6 billion of annual productivity improvements due to a more productive and engaged workforce;
- $9 billion in net health benefits by lowering the annual incidence of chronic disease and early mortality;
- more children who learn better and have higher educational outcomes and lifetime earnings—around $1.5 billion each year and;
- stronger and more cohesive communities resulting in greater social capital.” (p. 6)

Westerbeek et al. (2019) also review that sport can offer benefits to not only direct participants but also all involved. More than 2 million Australians volunteer in recreation clubs and community sport and benefit from volunteering in various ways. Furthermore, Jenkin et al. (2016) note the significant social benefits that can be gained by individuals who volunteer in sporting clubs.

Various researchers highlight critical transitional life stages related to dropout or retention in sport and PA (Craike et al., 2009; Eime et al., 2013, 2020c). Participation patterns at these transition stages often differ between sport and other leisure-time PAs. Research shows that older adolescents move their participation away from organized, competitive sport toward non-competitive setting and modes and more individual types of PA (Eime et al., 2013). For sport participation to grow, the policy-driven (recruitment) narrative needs to change toward engaging people in leisure time PA. As noted earlier in this section, an ecosystem approach driving policy across the PA-sport spectrum needs to be developed rather than the fragmented silos that currently exist separating traditional club-based competitive sport from the growing commercial sport and well-being and community sport and leisure sectors. This is why we work toward an integrated PA–Sport Participation framework in this paper.

## The Pattern of Sport Participation Across the Lifespan

The role that sport plays in society continues to evolve, and this is reflected in recent sport policy (Department of Health, 2018). For example, over recent years, there has been an increasing shift from traditional organized and competitive club-based sport to less structured, non-competitive, and individual forms of sport and PA (Eime et al., 2013; Eime R. et al., 2015; Borgers et al., 2016; Harris et al., 2017). Market segmentation research highlights that participation in club-based sport is not of interest for many children and adults (Australian Sports Commission, 2013a,b). Furthermore, other research also demonstrates that sport is not an activity for everyone (some do not want to play, others have limited or no access) and that females, older adults, those who are married, or those who have a disability are less likely to play sport (Eime et al., 2018b). Recent research also highlights that while many children and adults play sport, many do so not in the traditional club-based settings (Eime et al., 2020b).

A major underpinning of the integrated conceptual framework that we present in this paper is the fact that participation in organized sport only applies to a minority of the whole population, especially when considering participation across the lifespan. If we look at community-level sport and, in this context, organized club-based sport, then the pattern of sport participation across the lifespan becomes clear (Eime et al., 2020a). Participation in sport is popular for children, but it then quickly drops off significantly during adolescence (Eime et al., 2016a,b; Kemp et al., 2018). Furthermore, few adults and very few older adults participate in sport (Eime et al., 2016a,b), and adults are more likely to be active through general PA pursuits (Hulteen et al., 2017). Across the lifespan, there are transitional life stages that have a large (negative) impact (Van Houten et al., 2019) on participation in sport. Even yearly cross-sectional analysis shows the drop-off in participation rates from a peak for ages 10–14 years and then a significant decline for ages 15–19, and after that, a continual decline with age. Advocacy for the development of retention strategies, in that regard, has been increasing, especially during adolescence and for females (Eime et al., 2016a,b; Eime et al., 2020c).

Many sport governing bodies collect participant registration data (annual), as government funding through sport policy directs them to increase total numbers. As such, sport organizations will focus on that measure. We will demonstrate that most sport participation models focus on the elite pathway but only very few sport participants will become elite athletes, and at some time in adulthood, most people will drop out of sport. Where most participation models are only geared toward elite sport pathways, a population-level focused model identifying transitions in and out of sport and PA will provide more holistic insights. It will provide policy makers with a comprehensive view and assist in the development of strategies toward higher population levels of PA across the lifespan. This would, for example, better facilitate Sport Australia's aim to be the world's most active and healthy sporting nation (Department of Health, 2018).

## Sport Policy—Less About Mass Participation, More About Elite Sporting Success

Green (2007) found that a policy focus on elite sport achievement had been at the forefront of countries such as Australia and Canada since the early 1980s and that in the United Kingdom, in the lead up to the London 2012 Olympic Games, the central government started to allocate significant (and disproportionate) funding for elite sport development. This is further confirmed in a 15-nation comparative study by De Bosscher et al. (2015) and by Westerbeek (2021), in that since the first value explosion (of media rights) in the sport industry, an increasing number of nations around the world have become involved in a global sporting arms race. Governments are investing heavily in elite sport athletes, coaches, facilities, and other soft and hard sport infrastructure, in order to successfully compete at the major global sporting events such as the Olympic Games or World Championships. Many national governments invest more in elite sport than in community or participation-based sport, even when elite athletes only represent a very small proportion of the total population. It can therefore be contended that in the context of globalization of sport, the major event industry has been and continues to be instrumental in driving the value creation and growth of the sport (entertainment) industry as a whole.

The commercial and profile objectives of elite sport investment are distinctly different from those of community participation in sport, and so are policies that are developed for individual or community-level participation, and high performance or elite sport (Sport New Zealand, 2015; Department of Health, 2018). At the community level, sport policy is about the encouragement of people to be(come) active through sport and engage in PA their whole lives, building and nurturing healthy individuals and communities. In Australia, up until recently, the Australian Institute of Sport, as part of Sport Australia, was in charge of the high-performance plan “The Winning Edge.” This strategic plan was developed to re-establish Australia as a top five Olympic (medal tally) nation, and the plan was mapped out for 2012–2022. The Australian Institute of Sport was set up in response to Australia's poor performance at the 1976 Montreal Olympic Games, where the country did not win a single gold medal. In a 15-nation comparative study on Sport Policy factors Leading to International Sporting Success (SPLISS), Australia was identified as one of multiple nations significantly prioritizing funding elite sport over community sport. Admittedly, the study also found that most countries that invested more than others in elite preparation also were more successful (De Bosscher et al., 2015). It has taken close to four decades for the Australian government to take a more balanced view of where to invest in the nation's sport delivery system with the launch of its *Sport 2030* strategic plan. Recently, Sport Australia has shifted its priorities and has moved away from an extreme focus on elite sport to incorporating a population-based holistic and lifespan approach to sport and PA. The expressed priorities are now to: build a more active Australia, safeguarding the integrity of sport, achieving sporting excellence, and strengthening Australia's sport industry. The specific targets set to achieve this are as follows: improving physical health, improving mental health and personal development, strengthening communities, and growing the economy (Department of Health, 2018).

In line with more recent definitions used in Europe, a shift in policy language can also be detected in Australia. The shift from traditional competitive and club-based sport now takes in “a broad range of physical activities including informal, unstructured activity such as walking, riding, swimming, and running as well as the traditional, structured sport and new and evolving sport and PA offerings such as mixed martial arts, “ninja” style obstacle courses and stand-up-paddle boarding” (Department of Health, 2018, p. 6). As outlined earlier in this paper, the rationale for striving to increase PA levels across the population is supported with evidence of ill health resulting from physical inactivity. The economic (and increasing) weight of chronic disease in Australia is linked to physical inactivity (Department of Health, 2018). However, to this day, no strategic PA and sport participation pathway model or framework has been developed as a guide toward increasing PA and sport participation across the lifespan. A possible explanation for this rather serious oversight can be the fact that in most sport organizations (community clubs, State and National sport governing bodies), participation pathways remain in place that favor advancement to non-professional, semi-elite, and elite competition structures. The focus in organized sport remains on winning and achieving sporting excellence or success, which also includes the requirement to develop (and favor) sporting talent (Department of Health, 2018).

Therefore, the aim of this paper is to propose a population-based integrated framework that incorporates sport participation and engagement in PA, to match societal change and the evolving sport policy mandate of the new “sport.” The elite focused models fail to capitalize on pursuing objectives that seek to increase physical literacy levels, which indirectly can lead to higher levels of PA and sport participation and improved individual and community health. Overall, we have placed this extension and integration of various domains in context of an “ecosystem” and lifespan approach to better accommodate for sport to be positioned as a form of leisure-time PA, and where opportunities or obstacles to engage in leisure-time PA are influenced by a wide spectrum of conditions and organizations.

In the next section, we discuss and critique sport development models and then explore the current sport participation landscape in Australia and how sport policy and participation has changed over time and does change across the lifespan. We refer to Eime and Westerbeek's (forthcoming) Sport Participation Pathway Model (SPPM), which illustrates sport participation trends and highlights the significant dropout of sport particularly during childhood and adolescence (Eime et al., 2020a). We incorporate existing theory and insights from the SPPM model into the conceptual development of our integrated PA and sport framework, the Physical Activity Sport Participation framework (PASP), which we propose can assist with connecting sport policy to developing and implementing population-based PA strategies. As will be outlined, our framework is strongly underpinned by the recent Keegan et al. (2019). Physical Literacy model. This model strongly emphasizes the importance of a holistic lifespan approach to developing physical literacy that will allow for physically literate populations to be more active. In the near future, strategies would then focus on increasing population-level PA levels, using sport as an important (culturally significant) platform and option to do so.

## “Sport for all” and Participation Models That Focus on Elite Outcomes

In the context of this paper, it is important to briefly introduce the concept of Sport-for-All. Willem et al. (2019) argue that Sport-for-All is more an ideology rather than a part of sport organizations' activities that can be operationalized. In other words, where most sport organizations would have policies and systems working toward participation in community or elite sport, few if any would drive their operations toward Sport-for-All. To that end, most organizations that are in the “business of sport” could be classified as being a (minor or major) provider of Sport-for-All, even if this is not expressed in vision statements or business objectives. The literature on Sport-for-All is substantial and outside the scope of this paper to be extensively reviewed. However, it is worth noting that our proposed model very much aims to express a Sport-for-All approach, rather than the quite limited scope that existing sport participation models present.

Historically, sport participation models or frameworks have been developed specifically as talent or elite development pathways. As such, they are not necessarily relevant at the population level or across the lifespan. For example, the Standard Model of Talent Development (SMTD) is a pyramid model depicting at its base school sport and physical education and at the top of the pyramid national titles (Tinning et al., 1993), and it has been referred to by others as a performance (talent) pathway model. The pyramid structure highlights that fewer and fewer people are represented when moving up the pyramid into elite sport (Bailey and Collins, 2013). Other participation pathway frameworks such as the Foundations, Talent, Elite, and Mastery (FTEM) also has a large emphasis on talent development and elite athletes (Sport Australia, n.d). The FTEM does illustrate that when people move out of the FTEM, they can engage in an active lifestyle and/or sport. Even globally popular models, such as the LTAD (Long-Term Athlete Development) model, that acknowledge the importance of developing physical literacy place this importance in context of “for the development of children into elite athletes” (Ford et al., 2011, p. 390).

It can therefore be argued that such models were mainly developed to represent sports talent development and elite player pathways. They were not developed as lifespan population-level sports participation models. However, we advocate the need for a population level, which takes a lifespan approach and illustrates the movement across sport and physical activities and illustrate that physical literacy is required as a foundation to an active population.

## Introducing the Foundations to the PASP Framework

Eime and Westerbeek's (forthcoming) SPPM depicts current sport participation patterns and trends and, in doing so, emphasizes the significant dropout of sport (Eime et al., 2020a). Furthermore, when progressing across age groups, the issue of (poor) retention is clearly apparent, and absence from it being a focus in most sport policy is exposed. In presenting our holistic conceptual framework, we take as point of departure that developing a high level of physical literacy is fundamental to maximizing the chance to lead a physically active life, in turn increasing the likelihood of sport becoming an enjoyable leisure-time activity. We propose that the PASP can be used to drive specific strategies to increase participation in the new “sport,” which includes all leisure-time PA. Importantly, longitudinal data are required to not only evaluate the policy in terms of increasing participation but also maximizing retention, noting that from a public health perspective, switching between types of activities and contexts is fine.

We are using the SPPM as input into the development of the PASP framework as it illustrates the patterns of sport participation across the age groups and, in doing so, highlights the issue of dropout in organized sport and the lack of sport policies' focus on retention (Eime et al., 2020a). The development of the SPPM is underpinned by research evidence. The most important findings that have been included in the PASP framework are that:

- Many pre-schoolers have been recruited into club-based sport; however, the majority drop out within a couple of years, and perhaps they are not developmentally ready for organized sport (Eime R. M. et al., 2015; Eime et al., 2018a, 2020c).
- Young children are often active, resulting from participation in non-organized PA or free play and they are more likely to engage in free play than organized sport.
- In schools, children have the opportunity to play sport in either formal sport competitions or as part of their physical education.
- Participation is very popular among children aged 10–14 years (Eime R. M. et al., 2015; Eime et al., 2018a).
- Many people drop out of sport, specifically during late adolescence (Eime et al., 2019; Mathisen et al., 2019).
- Elite sport participants make up a very small proportion of sport participants.
- Beyond the ages of 20+ few adults, and fewer older adults participate in club-based sport (Eime and Harvey, 2018).

## International Trend Analysis in Regard to Sport and PA Policy Integration

As noted earlier in this paper, internationally, there has been a move toward significant investment in elite sport-driven policy development (De Bosscher et al., 2015). However, most recently with clear indications that an obesity epidemic is descending on much of the developed and developing world, some (already successful sporting) nations are starting to rethink their national level of sport investments. Rather than almost exclusively focusing on elite or high-performance sport, nations like the Netherlands, the UK, Finland, and New Zealand are increasing their support toward stimulating PA through national policy, which includes funding support for increasing community-level sport participation (Westerbeek et al., 2019). For example, in the lead up to the London Olympic Games in 2012, the UK government invested significantly in the preparation of the UK's elite athletes, much like Australia did through the Australian Institute of Sport in preparation for the Sydney Olympic Games in 2000. However, in 2015, the policy framework “Sporting Future: A New Strategy for an Active Nation” (UK Government, 2015) represented the most important shift in more than a decade for UK sport policy. This most recent policy document redefined what success would look like in relation to sport and it redirected focus from elite preparation to community engagement for population groups that are not engaged or have low engagement in sport participation. There are five key outcomes that were identified: individual development, physical well-being, social and community development, mental well-being, and economic development.

The Ministry of Health, Welfare and Sport in the Netherlands is in charge of developing and executing sport policy. Local governments mostly implement sport policy with the national government's role being primarily coordination. Although the Dutch government has always invested significantly in community-based sport delivery (above average compared to other nations), there is an increasing recognition that government investment is crucial to facilitate engagement in an active and healthy lifestyle (Waardenburg and van Bottenburg, 2013).

In September 2018, the Dutch government published “Plezier in bewegen” (“It is fun to move”), an advisory report produced by the Council for Community Health and Society, the Dutch Sport Council, and the Education Council (Dutch Sports Council, 2018). There are three key recommendations: to create and increase the opportunities for specialist physical education teachers to deliver programs, and assist and support other teachers to deliver such programs; to strengthen the engagement of primary to secondary school students in moderate-intensity PA to twice a day for at least half an hour; and to anchor and extend the programs delivered at the schools in the wider (local) community and appoint specialist coordinators to create and maintain a connected local network.

In Finland, “sport policy is designed to promote sport and PA and, through them, the wellbeing of the population, as well as competitive and performance sports and related civic activity” (Ministry of Education and Culture, n.d). Sport and PA contribute to a healthy population and are recognized as important factors for inclusion and in strengthening civil society (Prime Minister's Office Finland, 2015). In 2013, the Finnish Parliamentary Finance Committee recommended to focus sport funding on areas that produce the biggest increases in PA (Ministry of Social Affairs Health, 2013). In the “On the Move” National strategy for PA promoting health and well-being 2020, this recommendation was endorsed.

The Sports Act, introduced in 2014, already focused on sport participation for all in Finland (World Health Organization Regional Office for Europe, 2013). It confirms that sport and PA should be accessible to all citizens, and in the Act, population health and well-being finds its place next to elite sport.

The earlier noted major policy document is “On the Move.” “On the Move” is underpinned by the principles of a “Sport-for-All” policy and aims to promote engagement in PA for health and well-being for all Finnish citizens for the year 2020 (Ministry of Social Affairs Health, 2013). As we have noted earlier in regard to Sport-for-All, the Finish example shows how this ideological movement can influence broad-based policy development.

Sport New Zealand promotes, encourages, and supports sport and physical recreation across the country. Established in 2002 under the Sport and Recreation New Zealand Act 2002, Sport New Zealand has delivered three significant sport policy documents: the Sport NZ Group Strategic Plan 2015–2020; the Community Sport Strategy 2015–2020; and the High Performance Sport New Zealand Strategy 2017–2020.

Next to a specific high-performance strategy, the policies place strong emphasis on strengthening the local delivery of sport across the life course, with special focus on communities in which there are low levels of participation. The Sport NZ Group Strategic Plan recognizes the ability of grassroots sport participation to add significant value to lives and provide entry points into high-performance sport. There are four focus areas guiding investment and resources in order to create pathways toward participation but also what is required to win on the world stage: young people, competitive sports (including talent identification), local delivery (particularly in low-participation communities), and leading high performance.

Sport New Zealand also produced “The Value of Sport” (Angus and Associates, 2017), which is a comprehensive outlook of the health, developmental, social, economic, and overall well-being benefits of sport. It reports the significant health benefits of investing in sport to address increasing frequencies of chronic disease in New Zealand. It was estimated that in 2013, NZ$200 million of direct health care costs could be attributed to physical inactivity. It states that, if physical inactivity was eliminated, several preventable chronic diseases could be largely be evaded including 7.9% of heart diseases, 9.8% of Type 2 diabetes and more than 13% of breast cancer cases (Ding et al., 2016).

Many nations have populations that are largely inactive, and this places a great burden on the health system and as such on the economy as a whole. Internationally, sport policies are becoming more consistent in defining sport as a continuum from recreation-based leisure-time PA such as walking, to competitive club-based sport, and then back to more recreation-based sport and PA. However, PA in general is void of a structured “delivery system” and organized competitive sport is isolated within individual sports organizations with a large emphasis on elite performance and elite development. Eime and Westerbeek (forthcoming) demonstrated that sport policy focusing on increasing participation numbers may not be the most effective avenue toward stimulating lifelong active lifestyles as most people will soon(er) (rather than later) drop out of sport (Eime et al., 2020a). It seems odd that sport organizations do not pay more strategic attention to retaining active participants. Backed by recent research, we have argued that sport in its competitive, traditional club-based form of activity is not a population-based setting for participation (Eime et al., 2020b), and therefore cannot be left with the huge responsibility to tackle population levels of physical inactivity. To that end, we have positioned sport as part of a larger ecosystem that centers on lifelong engagement in PA, at a population level. This will allow for better positioning of sporting clubs, the role they can play, and impact that they can have on facilitating higher levels of PA across the lifespan. We have captured this in a conceptual model that we will next introduce as the PASP framework. To our knowledge, this will be the first time that a framework has been developed that maps sport participation across the whole leisure-time PA spectrum and across the lifespan, and that is underpinned by the need to develop high levels of individual physical literacy.

## The Physical Activity and Sport Participation (PASP) Framework

We have developed a framework founded on relevant research evidence that reflects the current (international) policy transition toward increasing population levels of PA (rather than focusing on elite sport performance outcomes and with a predominant policy focus on increasing sport participation). Sport, as outlined in this paper, plays an important role in that regard, but cannot be left with the sole responsibility to achieve population-wide recommended levels of PA. As a matter of fact, as is pictured in our framework, higher participation in sport will be an outcome of improving people's physical literacy and increasing their overall PA levels. Only then will sport participation increase and endure across the lifespan. Our framework is further premised on a number of evidence-based insights that were presented earlier in this paper. These are:

- That developing physical literacy (Whitehead, 2010) is foundational and critical to living an active lifestyle;
- That parents, physical education teachers, and the wider (PA ecosystem) environment are important facilitators of developing physical literacy;
- That development of people's fundamental movements skills is critical (the physical component of physical literacy), but for people to become (holistically) physically literate, developing the psychological, cognitive, and social elements is equally important;
- That there are critical transition points during early life stages for sport policy makers to consider. Every stage requires a different policy focus, and these move from offering modified sport as a means to recruiting very young children into sport, to retaining adolescents and adults as sport participants, to retaining the elderly as actively involved and engaged in sport clubs;
- That there are three major life stages in regard to developing or maintaining high levels of PA that in regard to the intent of being physically active broadly range from a *preventative*, to *maintenance*, and then a *curative* approach with advancing age;
- That a good level of physical literacy mostly leads to high levels of PA (active lifestyle) and that a poor level of physical literacy mostly leads to low levels of PA (sedentary lifestyle).

**Figure 1:**
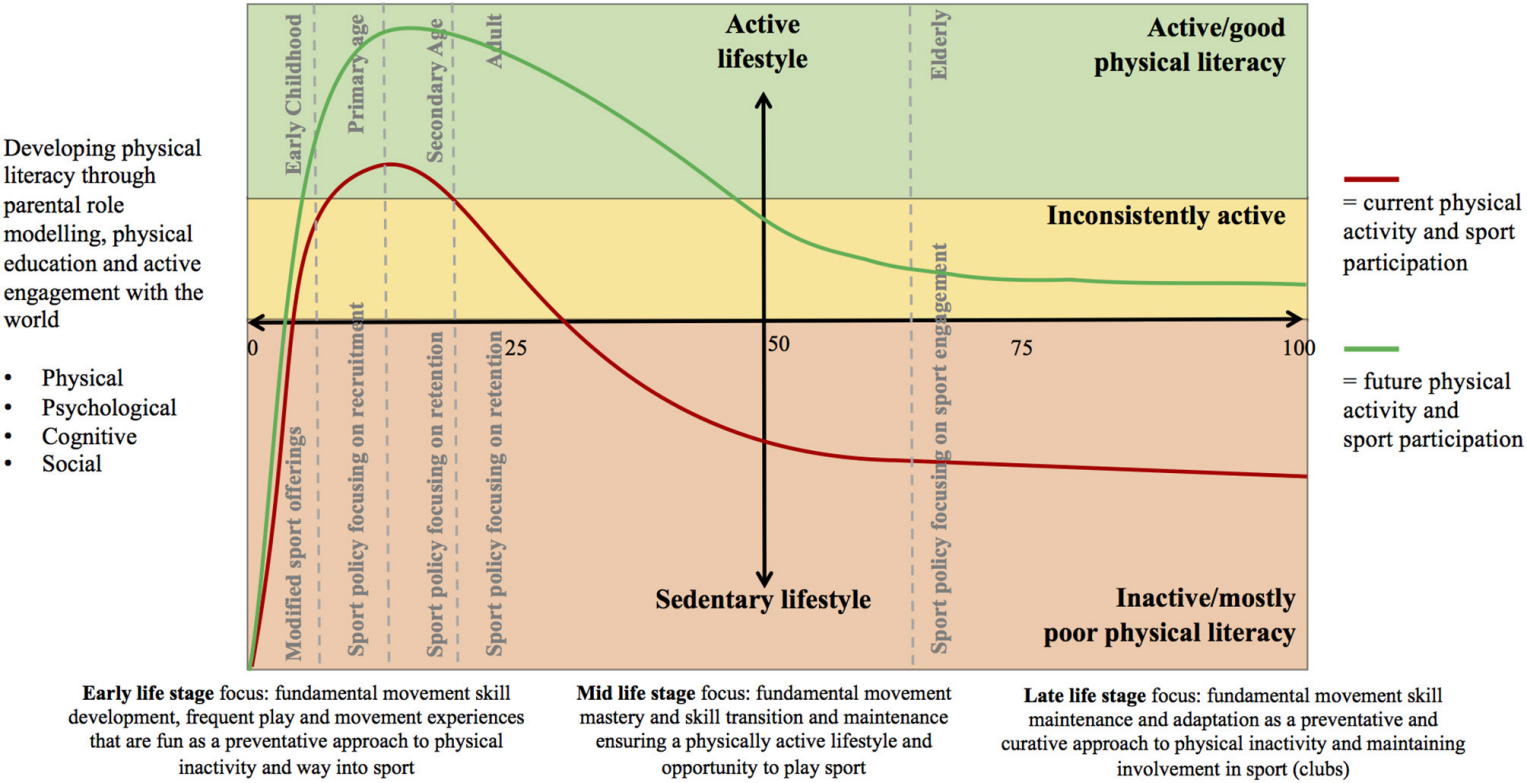
The Physical Activity and Sport Participation (PASP) framework.

We then present two trend lines across the lifespan: a red line that highlights estimated current levels of PA and sport participation and a green line that envisions (aspirational) future levels of PA and sport participation. The PASP framework is presented in Figure 1. The framework can be best understood by moving from left to right—as we age from young to old. It depicts various life stages that are separated by developmental PA transition points as evidenced by research. These stages are early childhood, primary age, secondary age, adult, and elderly. Each of these stages requires policy makers and strategies to focus on what is most likely to bring or keep people into sport. These foci move from modified sport offerings, to recruitment, to retention, to engagement. Underpinning the whole model is a lifelong commitment to developing and maintaining a high level of physical literacy, which, in turn, would positively impact the likelihood of people becoming and remaining physically active, which, in turn, provides fertile ground for sport organizations to recruit physically more active people into sport. Early engagement in PA and sport is a preventative approach to becoming physically inactive as higher levels of physical literacy and activity increase the chances of more active (maintenance) lifestyles. As noted before, we have mapped the estimated population level of PA and sport across the lifespan with the red line (too few people are sufficiently active, in particular after entering adulthood). We propose that the PASP framework, when considered as a tool for policy development in sport and PA, will lead to outcomes that allow for the drawing of the aspirational green line, where the majority of the population engages in sufficient levels of PA and sport.

## Conclusion

Patterns of participation in leisure-time PA and sport differ considerably across the lifespan in terms of the types of activities and settings of and motivations for participation and are further influenced by changes in society and the policy context in which sport is produced, delivered, and consumed. Historically, sport policy largely focused on competitive club-based sport and elite performance. In line with this, talent development pathway models were developed and implemented.

At a population level, competitive club-based sport alone is not going to solve the physical inactivity crisis, as evidenced by the population trends in participation in sport and the high dropout of competitive club-based sport. Internationally, sport policies are transitioning toward integrating sport in the leisure-time PA continuum (we have proposed an ecosystem approach), which also extends sport beyond the traditional club-based system. Therefore, we have developed a population-based, whole of “sport” and PA lifespan framework, PASP.

The basic logic underpinning our framework reflects the emerging policy environment that emphasizes increasing population PA levels. First, policy needs to focus on maximizing individuals' opportunities to develop a good level of physical literacy during childhood, as a foundation for lifelong activity. As frequent and competent human movement is critical throughout the lifespan, a range of policy levers can be used to further facilitate such opportunities, for example, by the development of play or work environments that encourage people to be physically active, or by increasing quality physical education classes in primary and secondary school curricula. For sport organizations, the opportunities are obvious. A larger pool of potential participants will become available when levels of skill mastery and movement confidence increase. Sport governing bodies and sport clubs have to become smarter and more strategic in their marketing and service offering focus, in that there is clear evidence of transition points during the early life-stages about the type of sport offerings (potential) participants want, and how this affects the extent and duration of their membership-based involvement.

We propose that the PASP framework can serve as a holistic policy and implementation model for both the whole of sport including governmental PA and sport policy makers, sport governing bodies and clubs, the ever-increasing range of private PA and sport providers, and health (promoting) agencies. The “how,” “who,” “what,” and “when” questions are relevant but beyond the scope of this paper. We hope that the framework can guide in answering some of those questions. It remains critical in that regard, that measurement of participation and retention in combination with consumer behavioral data is used to inform and evaluate policies and strategies. In recognition of the changing patterns of participation in sport and PA across the lifespan, the PASP framework can contribute to coordinated and integrated PA and sport policy development, which, in turn, can lead to strategies that tackle the global physical inactivity crisis. Sport will remain an important player in that regard.

## Author Contributions

HW and RE have jointly developed and authored this paper. They agree to be accountable for the content of the work. All authors contributed to the article and approved the submitted version.

## Conflict of Interest

The authors declare that the research was conducted in the absence of any commercial or financial relationships that could be construed as a potential conflict of interest.
